# Endurance exercise is a leptin signaling mimetic in hypothalamus of Wistar rats

**DOI:** 10.1186/1476-511X-10-225

**Published:** 2011-12-02

**Authors:** Jiexiu Zhao, Ye Tian, Jincheng Xu, Dongsen Liu, Xiaofang Wang, Binxiu Zhao

**Affiliations:** 1 Sport Biological Center, China Institute of Sport Science, Beijing, 100061, PR China; 2 Departments of Kinesiology, Beijing Sport University, Beijing, 100084, PR China; 3 Department of Biomedical Sciences, Baylor College of Dentistry, Texas A & M University System Health Science Center, Dallas, TX, 75246, USA; 4 First People's Hospital of Shijiazhuang, Hebei, 050011, PR China

**Keywords:** TREADMILL, JAK2, STAT3, Akt, ERKs, SOCS3, IL-6

## Abstract

**Background:**

Endurance exercise is known to promote a substantial effect on the energy balance in rats and humans. However, little is known about the exact mechanisms for the appetite-suppressive effects of endurance exercise. We hypothesized that endurance training might activate signaling cascades in the hypothalamus known to be involved in leptin signaling.

**Methods:**

16 male Wistar rats were randomly assigned to two groups: sedentary (n = 8) and exercise groups (n = 8). Animals in the exercise group started treadmill running at 30 m/min, 0% grade, for 1 min/bout. Running time was gradually increased by 2 min/bout every day. The training plan was one bout per day during initial two weeks, and two bouts per day during 3rd-9th week. At the end of nine-week experiment, blood was analyzed for low-density lipoprotein cholesterol (LDL-C), triglyceride (TG), total cholesterol (TC), free fatty acid (FFA), interleukin (IL)-6, and leptin in both groups. Activations of janus kinase 2-signaling transducer and activator of transcription 3 (JAK2-STAT3), protein kinase B (Akt), extracellular regulated kninase (ERKs), and suppressor of cytokine signaling 3 (SOCS3) in hypothalamus were measured in the end of nine weeks of exercise protocol.

**Results:**

Nine-week endurance exercise induced lower concentrations of LDL-C, TG, TC, FFA, and leptin in rats (*P *< 0.05 or *P *< 0.01). Nine-week endurance exercise significantly increased the circulating IL-6 concentration compared with sedentary group (239.6 ± 37.2 pg/ml vs. 151.8 ± 31.5 pg/ml, *P *< 0.01). Exercise rats showed significant increases in JAK2, STAT3, Akt, ERKs, and SOCS3 phosphorylations compared with sedentary rats (*P *< 0.01).

**Conclusion:**

The data suggest that endurance exercise is a leptin signaling mimetic in hypothalamus of Wistar rats.

## Introduction

There is an obesity epidemic in much of the developed and developing world [1-6]. Leptin is a cytokine originating mainly from white adipose tissue that plays an important role in regulating energy expenditure, food intake and obesity [7]. The mechanism by which leptin modulates these hypothalamic neurons involves the binding of leptin to the long form of leptin receptor (Ob-Rb) [8] and the subsequent intracellular signaling [9], initiated by autophosphorylation of Janus kinase 2 (JAK2) and activation of signal transducer and activator of transcription 2 (STAT3) [10]. After the translocation of STAT3 to the nucleus, suppressor of cytokine signaling-3 (SOCS3) is activated, exerting feedback inhibition on JAK2 [11]. Furthermore, leptin activation of insulin receptor substrates (IRSs) and the protein kinase B (Akt) pathway inhibits food intake [12] and modulation of extracellular regulated kinases (ERKs) has been shown to play a function in the control of energy homeostasis [13]. However, leptin administration to obese rats [14,15] and humans [16,17] has elicited small effects on fat mass and appetite due to leptin resistance. Endurance exercise of medium intensity is known to profoundly affect energy balance [18-20]. If endurance exercise is able to activate the same signaling cascades as leptin is remains to be elucidated.

The level of circulating interleukin-6 (IL-6) increases dramatically in response to endurance exercise [21,22], with IL-6 being produced by working muscle [23,24] and adipose tissue [25,26]. IL-6 seems to have several important roles in metabolism, including induction of lipolysis [25,27] and enhancement of insulin sensitivity when injected into IL-6-deficient mice [28]. Thus it is possible that the effects of endurance exercise on leptin signaling pathways in hypothalamus may be dependent on IL-6.

The role of endurance training as a modulator of leptin's action has not been completely examined [29,30]. Endurance trained humans possess a greater capacity to oxidize fatty acid while lower concentrations of circulating leptin [31]. Another study indicated that leptin levels either expressed in absolute or relative to adiposity values are decreased with treadmill training in female Sprague-Dawley rats [32]. Taken together, it seems that endurance exercise might improve leptin sensitivity. Previous study demonstrates that sprint exercise is a leptin signaling mimetic in human skeletal muscle. However, we do not know if endurance exercise has similar mechanism in rat hypothalamus.

Therefore, the main aim of the study was to determine if a nine-week endurance exercise may act as leptin mimetic, by examining the response of the known leptin signaling pathway in hypothalamus. Another aim was to determine if nine-week endurance has positive effect on blood lipid profiles in rats. The hypothesis to be tested was that nine-week endurance exercise will activate signaling cascades in the hypothalamus known to be involved in leptin signaling pathways and that this effect will occur independently of circulating leptin concentrations.

## Materials and methods

### Animals

Sixteen male Wistar rats (aged 2 months), was supplied by the Animal House of the Chinese Academy of Medicine, weighing 203 ± 15 g were used in this experimental study. All rats were given standard rat food and tap water ad libitum and housed at 23 ± 2°C on a 12:12-h dark-light cycle. The animals were divided into two groups: sedentary group (n = 8) and exercise group (n = 8). Care and procedures were based on the guidelines of the National Institute of Health (NIH), and was approved by the local Animal Care and Usage Committee.

### Experimental protocol

Rats in the exercise group were introduced to running on a motor driven rodent treadmill (BCPT-96, Hangzhou, China). The treadmill was equipped with an electric shocking grid on the rear barrier to provide exercise motivation to the rats. Animals of exercised groups started treadmill running at 30 m/min, 0% grade, 1 min/bout. Running time was gradually increased by 2 min/bout every day. Exercise intensity of the endurance program was about 65% of maximal oxygen consumption according to previous study of oxygen consumption during a progressive exercise test in rats [33]. At the end of the nine-week experiment, the rats of exercise group were anesthetized with an intraperitoneal injection of pentobarbital sodium, 40 mg/kg of body weight, at 12 h after the last training. The rats of control group were anesthetized in the same time of exercise group. Epididymal, retroperitoneal, perirenal, mesenteric, andinguinal adipose depots were removed and weighed.

### Biochemical measurement

At the end of experiment, blood samples were collected from abdominal aorta and drawn into capillary tubes, sealed, stored on ice, centrifuged immediately after the test, and stored at -20°C for determination of related indexes. High-density lipoprotein cholesterol (HDL), low-density lipoprotein cholesterol (LDL), free fatty acid (FFA), triglyceride (TG), total and cholesterol (TC) levels were determined using commercially available kits for rats (Nanjing Jiancheng Bioengineering Institute, China). Serum leptin level was determined by radioimmunoassay kit for rats (Linco Research, St-Charles, Missouri, USA). Level of IL-6 in serum was measured with commercial enzyme-linked immunosorbent assay (ELISA) kits following the instructions of the manufacturer (RapidBio Lab, CA, USA).

### Western blot analysis

The hypothalamus from exercise and sedentary groups were lysed in a lysis buffer containing 20 mM Tris (Ph 7.4), 2 mM EDTA, 137 mM NaCl, 1% NP40, 10% glycerol, 12 mM α-glycerol phosphate, and supplemented protease inhibitors [34]. Protein concentration of the supernatant was measured by Bio-Rad protein assay kit (Bio-Rad Laboratories, Hercules, Calif., USA). Equal amounts of protein were used for immunoprecipitation followed by Western blot analysis with the indicated antibodies and ^125^I-protein A. ^125^I-protein A bound to anti-peptide antidodies was detected by autoradiography, using preflashed Kodak XAR film (Eastman Kodak, Rochester, NY) with Cronex Lightning Plus intensifying screens at -80°C for 24-48 h. Band intensities were calculated by optical densitometry of the developed autoradiographs. Antibodies for phosphorylated JAK2, STAT3, Akt, ERKs (ERK1/2), and SOCS3 were from Santa Cruz Biotechnology (Santa Cruz, CA).

### Quantitative real-time PCR

Total RNA was extracted from Arcuate Nucleus (ARC) of the hypothalamus by using the single-step, acid guanidium thiocyanate, phenol-chloroform extraction as described by previous study [35]. The primer and the TaqMan probe for rat Ob-Rb mRNA were designed using Primer Express software (Applied Biosystems). The nucleotide positions of the oligonucleotides were as follows. Rat's Ob-Rb (GenBank Accession No. U52966): forward primer 5'-AAAGCCTGAAACATTTGAGCATC-3', reverse primer 5'-CCAGAAGAAGAGGACCAAATATCAC-3'. The prime were searched by an NCBI BLAST homology search to ensure that they were specific for the target mRNA transcript and species. The primer of rat β-actin (internal standard) used in the real time-PCR sequences according to previous paper [36]. The real time PCR assay was performed in 50 μl of Taqman One-Step RT-PCR Master Mix reagents containing 300 nM forward primer, 900 nM reverse primer, 200 nM TaqMan prober, and 20 ng of total RNA. The assay was carried out using the ABI PRISM 7300 Sequence Detection System (Apllied Biosystems) under condition as follow: 95°C for 2 min, followed by 45 cycles of 95°C for 25 s, at 60°C for 25 s, and at 72°C for 40 s. The relative expression of each mRNA was calculated as the ΔC_t _(the value obtained by subtracting the C_t _value of the β-actin from the C_t _value of the Ob-Rb mRNA).

### Statistical analyses

Comparisons of body weight, food intake and fat pads in different groups were carried out using repeated measures ANOVA. Independent Student's t-test was used to analyze group difference in other variables. The results were expressed as means ± SD. The statistical calculations were performed using SPSS software for windows (version 16.0). A probability value of *P *< 0.05 was taken to be statistically significant.

## Results

### Effect of endurance exercise on body weight, food intake

**Figure 1:**
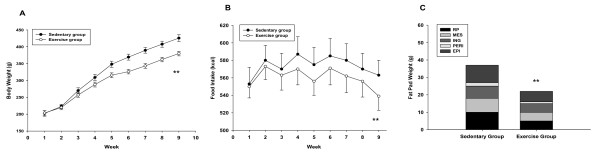
**Effects of endurance exercise on the body weight (A), food intake (B), and fat pad weight (C). EPI, epididymal; RP, retroperitoneal; PERI, perirenal; MES, mesenteric; ING, inguinal**. Significant exercise effect by repeated measures ANOVA: ** *P *< 0.01.

Over the full nine-week endurance exercise, exercise rats gained about 10% less body weight than did sedentary rats (Figure 1). Furthermore, nine-week endurance exercise led to a 40% reduction in total fat pad mass (Figure 1). Exercise rats failed to increase their caloric intake to compensate for their increased energy expenditure either during or after exercise termination (Figure 1).

### Effect of endurance exercise on serum LDL-C, TG, TC, FFA, and leptin

Nine-week endurance exercise induced lower concentrations of LDL-C, TG, TC, FFA, and leptin compared with sedentary rats (*P *< 0.05 or *P *< 0.01, Table 1). However, the serum IL-6 concentration was higher in the exercise group compared with sedentary group (239.6 ± 37.2 pg/ml vs. 151.8 ± 31.5 pg/ml, *P *< 0.01, Table 1).

**Table 1:** Serum indices of lipid metabolism in sedentary and exercised rats.

	Sedentary Group	Exercise Group
HDL-C, mmol/l	1.25 ± 0.43	1.76 ± 1.07
LDL-C, mmol/l	2.05 ± 0.35	1.74 ± 0.25 *
TG, mmol/l	0.94 ± 0.22	0.86 ± 0.15 *
TC, mmol/l	0.82 ± 0.20	0.77 ± 0.19 *
FFA, μmol/l	267.9 ± 62.8	134.1 ± 45.6 **
leptin, ng/ml	3.7 ± 0.8	2.1 ± 0.7 **
IL-6, pg/ml	151.8 ± 31.5	239.6 ± 37.2 **

Values are means ± SD. HDL, high-density lipoprotein cholesterol; LDL, low-density lipoprotein cholesterol; TG, triglyceride; TC, total cholesterol; FFA, free fatty acid; IL-6, interleukin-6. Significant difference from sedentary group, * *P *< 0.05, ** *P *< 0.01.

### Effect of endurance exercise on signaling cascades in hypothalamus

**Figure 2:**
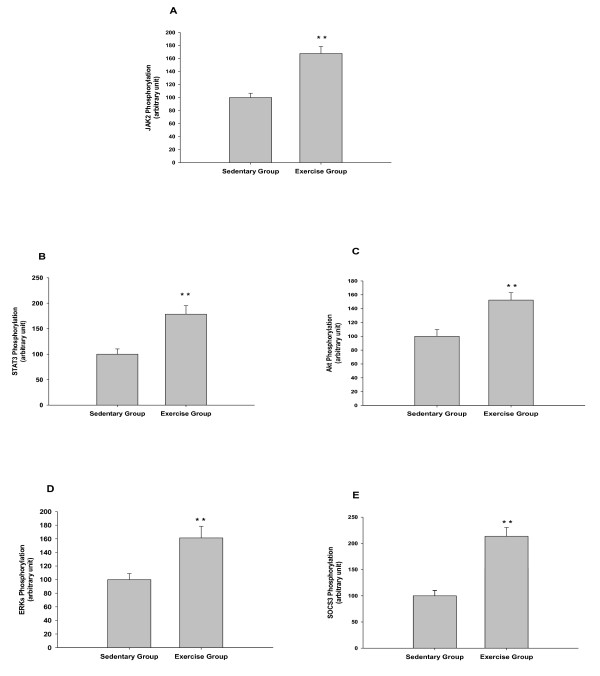
**Effects of endurance exercise on the signaling cascades in hypothalamus. (A) JAK2 phosphorylation concentrations in exercise and sedentary groups. (B) STAT3 phosphorylation levels in exercise and sedentary groups. (C) Akt phosphorylation levels in exercise and sedentary groups. (D) ERKs phosphorylation levels in exercise and sedentary groups. (E) SOCS3 phosphorylation levels in exercise and sedentary groups**. Signaling phosphorylations were normalized to total signaling, and levels in sedentary rats were set to 100, with SD adjusted proportionally. ** *P *< 0.01 vs. sedentary rats.

Endurance exercise enhanced JAK2 phosphorylation in the hypothalamus (167.5 ± 10.9 vs. 100.0 ± 6.6 arbitrary units, for exercise vs. sedentary rats, *P *< 0.01, Figure 2A). Exercise group had higher STAT3 phosphorylation level than sedentary group (178.5 ± 16.7 vs. 100.0 ± 10.2 arbitrary units, for exercised vs. sedentary groups, *P *< 0.01, Figure 2B). As expected, phosphorylated Akt was increased significantly in exercise group compared with sedentary group (152.3 ± 10.7 vs. 100.0 ± 9.8 arbitrary units, for exercised vs. sedentary groups, *P *< 0.01, Figure 2C). ERKs activation was higher in exercise group compared with sedentary group (161.3 ± 17.1 vs. 100.0 ± 8.9 arbitrary units, for exercised vs. sedentary groups, *P *< 0.01, Figure 2D). In addition, exercise group had higher SOCS3 phosphorylation levels that sedentary group (213.5 ± 16.7 vs. 100.0 ± 10.3 arbitrary units, for exercised vs. sedentary groups, *P *< 0.01, Figure 2E).

**Figure 3:**
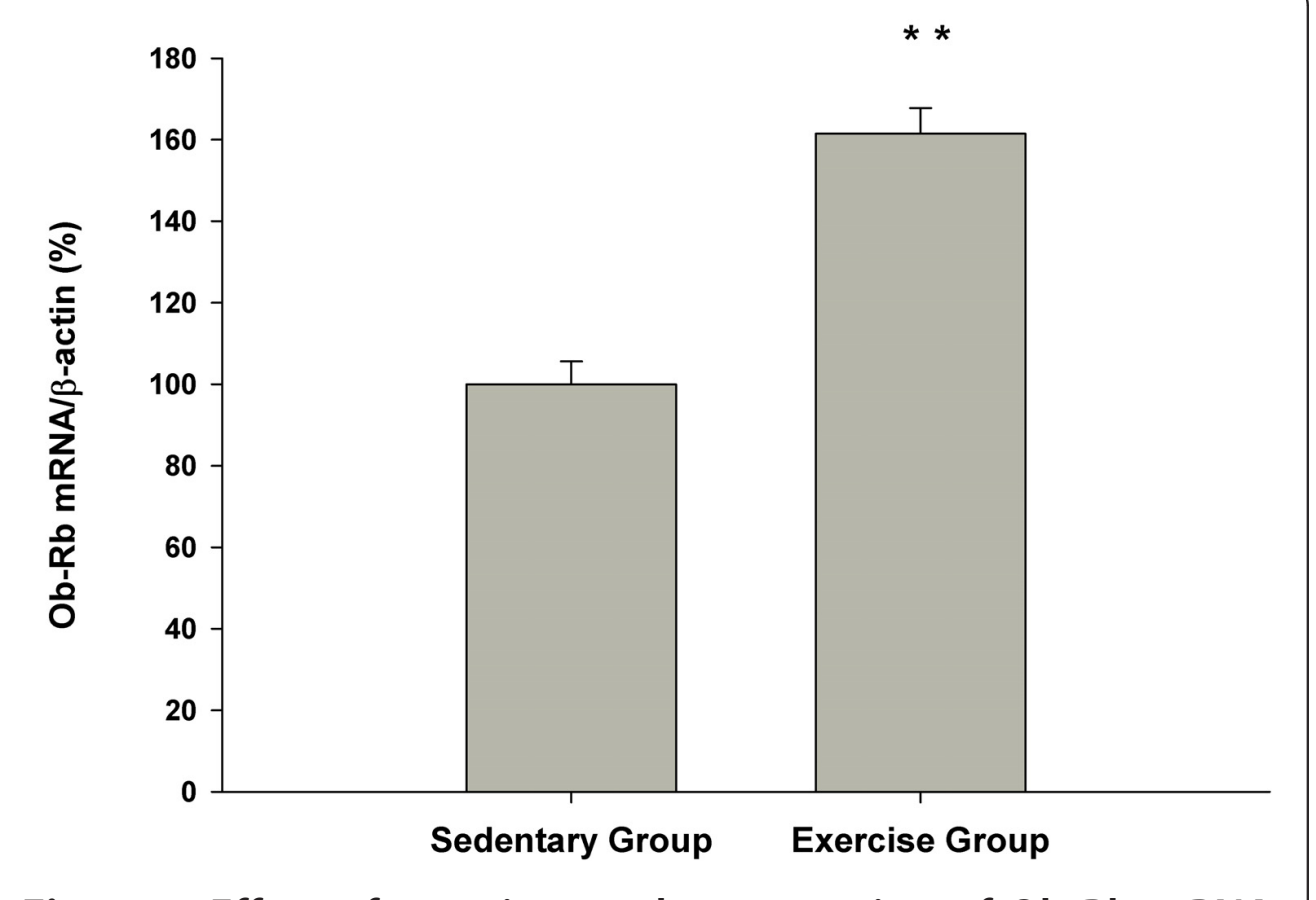
**Effect of exercise on the expression of Ob-Rb mRNA quantified relative to β-actin in hypothalamus**. Concentrations in sedentary rats were set to 100, with SD adjusted proportionally. ** *P *< 0.01 vs. sedentary rats.

Rats in the exercised group demonstrated significant higher (+60%) than sedentary group in Ob-Rb mRNA concentration (161.5 ± 6.3 vs. 100.0 ± 5.6 arbitrary units, for exercised vs. sedentary groups, *P *< 0.01, Figure 3).

## Discussion

The present study examined changes in leptin signaling cascades in hypothalamus of Wistar rats. The major finding of this study is nine-week endurance exercise resulted in significant decreases in the body weight, food intake, and fat pad weight (Figure 1). In agreement with our hypothesis, nine-week endurance exercise increased Ob-Rb mRNA expression (Figure 3) and signaling cascades (Figure 2) in the hypothalamus compared with sedentary group. The changes in signaling pathways occurred with same changes in circulating IL-6 concentrations in exercise rats. However, circulating leptin concentration was decreased in exercise group compared with sedentary group (Table 1). Altogether, these results indicate that nine-week endurance performed leptin mimetic in hypothalamus of rats.

### Effect of endurance exercise on fat mass and serum LDL-C, TG, TC, FFA

The present study indicated that endurance exercise induced reduction in fat mass in rats compared with sedentary rats. This results is similar to previous reports in rats [37] and mice [38]. Along with fat mass indexes, we monitored the major parameters of lipid metabolism. The results showed that concentrations of LDL-C, TG, TC, and FFA decreased significantly after endurance exercise (*P *< 0.05 or *P *< 0.01). This finding is in line with other endurance training and FFA studies, showing that endurance training results in significant improvements in FFA mobilization and oxidation [39]. Therefore, it seems that endurance exercise has positive effect on adipose tissue metabolism in rats.

### Effect of endurance exercise on signaling cascades in hypothalamus

There is recent evidence that hypothalamic leptin signal pathway and Ob-Rb are reliable indexes to reflect adiposity levels [40,41]. However, the effect of endurance exercise on hypothalamic leptin signaling and Ob-Rb mRNA is unknown. In an effort to fill this gap, the present work measured seven biological parameters (with the emphasis on leptin sinaling and Ob-Rb mRNA) after nine-week endurance exercise. In contrast to non-Arcuate Nucleus (ARC) long form Ob-Rb neurons, ARC long form Ob-Rb neurons might directly access circulating leptin [42]. We can predict that the ARC is an excellent sensor of peripheral metabolism of leptin. In this context, it seems that ARC of the hypothalamus is one of the key organs involved in leptin action. Our data show that Ob-Rb mRNA of hypothalamus ARC was increased significantly and circulating leptin was decreased significantly by the endurance exercise. The results support previous hypothesis that training by decreasing plasma leptin levels may favor the peripheral action of leptin through the hypothalamic centers [43]. These findings agree with another study [43,44]. They found that endurance exercise induced decreases in plasma leptin levels is accompanied by a reduction in gene expression of leptin receptors in liver. Based on the information, it is possible that endurance exercise reinforces the central rather than peripheral action of leptin. On the other hand, one study showed that muscle hypertrophy and increased expression of leptin receptors in the musculus triceps brachii of the dominant arm in professional tennis players [45]. It seems to us that conflicting results in the literature may stem from two sources primarily. One is intrinsic limitation of different subjects and different tissues of the Ob-Rb mRNA. The subjects in study of Olmedillas et al. are tennis players and muscle tissues were measured for Ob-Rb mRNA. However, we chose rats as subjects and hypothalamuses were measured for Ob-Rb mRNA. Another is different exercise mode. Endurance exercise was taken in present study, while tennis was taken in the study of different results [45]. However, it is imperative that future studies examine the expression of the Ob-Rb protein in hypothalamus and its relationship to leptin singing pathway.

Regulation of energy balance is an essential function of the human organism that is controlled by the central nervous system and an elaborate interplay of intertissue signaling [44]. IL-6 is one peptide hormone that could supply peripheral feedback to the hypothalamus [44], and contributes to substrate availability and utilization by facilitating an increase in glucose and lipid metabolism to maintain metabolic homeostasis during endurance exercise [46]. IL-6 peripheral signal activates several hypothalamic hormonal pathways during training [47]. It is clear that during endurance exercise, IL-6 can be produced and released from skeletal muscle tissue [48]. This is likely the reason why the circulating IL-6 is increased after nine-week endurance exercise in our study.

Our data provide evidence that there is an increase in the JAK2, STAT3, Akt, ERKs, and SOCS3 signaling cascades (Figure 2). Leptin activation of STAT3 requires the leptin receptor, which associates with and activates JAK2 in a ligand-dependent manner [49,50]. The current study provide direct measurements of leptin signaling in the hypothalamus after endurance exercise, and it documents increased sensitivity to JAK2/STAT3 pathway in the hypothalamus of exercised rats. ERKs may be phosphorylated directly by JAK2, i.e., by an OB-Rb-independent mechanism [51]. Our results are in line with previous studies that endurance exercise can induce ERKs phosphorylation [52-54]. Endurance exercise could increase SOCS3 expression in rats and the change has potential relationship to IL-6 expression [55]. Our results also imply that increased SOCS3 is caused by higher IL-6 concentrations induced by endurance exercise. Little is known about the potential effect of endurance exercise on Akt. These finding provides support for the hypothesis that endurance exercise could have appetite-suppressive actions mediated by the hypothalamus. Although JAK2, STAT3, Akt, ERKs, and SOCS3 are signaling cascades of leptin, decreased leptin concentrations and increased signaling cascades after nine-week endurance exercise implying that circulating leptin could hardly explain the observed responses of signaling phosphorylations in hypothalamus. Higher increases in circulating IL-6 are needed to induce STAT3 and SOCS3 phosphorylation [55,56]. It is possible that increased circulating IL-6 could easily explain the increased singling levels in hypothalamus of exercise rats compared with sedentary rats.

In conclusion, this study demonstrates that the signaling pathways activated by leptin in hypothalamus are also activated by nine-week endurance exercise in hypothalamus of Wistar rats, despite a reduction of leptin serum concentration after the endurance exercise. These finding imply that endurance behave as a leptin mimetic and could be used to stimulate the leptin signaling pathway in haypothalamus of Wistar rats. This opens the possibility of using endurance exercise to circumvent leptin resistance in obese humans and rats and may lead to increased leptin sensitivity. We provide some evidences to support that the effects of endurance exercise on JAK2, STAT3, Akt, ERKs, and SOCS3 are mediated by change in serum IL-6 concentration.

## Competing interests

The authors declare that they have no competing interests.

## Authors' contributions

JXZ and YT have conceived the study and its design and obtained research grants for this study. JCX, DSL, JXZ, XFW, and BXZ have analyzed the date, interpreted and written the final draft of this manuscript. All authors have read and approved the final manuscript.
